# Escort Aptamers: New Tools for the Targeted Delivery of Therapeutics into Cells

**Published:** 2011

**Authors:** A.S. Davydova, M.A. Vorobjeva, A.G. Venyaminova

**Affiliations:** Institute of Chemical Biology and Fundamental Medicine, Siberian Branch, Russian Academy of Sciences

**Keywords:** SELEX method, NA aptamers, escort aptamers, specific cell binding, addressed cell delivery, detection of cells

## Abstract

Escort aptamers are DNA or RNA sequences with high affinity to certain cell-surface proteins, which can be used for targeted delivery of various agents into cells of a definite type. The peculiarities of the selection of escort aptamers are discussed in this review. The methods used in selection of escort aptamers via the SELEX technique are considered, including selection against isolated cell-surface proteins, cell fragments, living eukaryotic cells, and bacteria. Particular attention is given to the design and chemical modification of escort aptamers. The different fields of application of escort aptamers are described, including the targeted delivery of siRNAs, nanoparticles, toxins, and photoagents, as well as the identification of specific cell markers and the detection or isolation of cells of a definite type. The potential for the application of escort aptamers in the development of new therapeutic agents and diagnostic systems is also discussed.

## INTRODUCTION

**Fig. 1:**
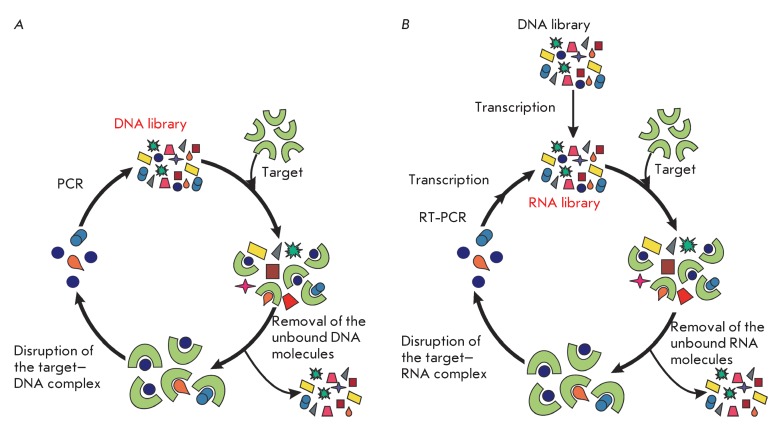
The general scheme of the SELEX method using DNA (A) and RNA (B) libraries.

Aptamers (Latin *aptus*  – suitable) are single-stranded DNA and RNA molecules that are capable of specific recognition of definite types of compounds, thanks to their unique spatial structure. In the 1990s, methods for *in vitro * selection, enabling one to obtain nucleic acids with predetermined properties, were described by three independent research groups. A. Ellington and J. Szostak [1] obtained an RNA molecule that was capable of specifically binding to an organic dye. C. Tuerk and L. Gold described the selection of RNA molecules that were capable of binding to phage 4 DNA polymerase and called the developed method SELEX (Systematic Evolution of Ligands by Exponential Enrichment) [2]. D. Robertson and G. Joyce used *in vitro * selection to convert a group I ribozyme from a ribonuclease into a deoxyribonuclease [3]. Throughout the subsequent two decades, this field has developed rapidly; methods for the selection of aptamers and approaches to their design have been further refined. A large number of aptamers capable of binding to various targets with high specificity have already been obtained (see Reviews [4–7]). Aptamers find broad application across a wide range of research fields, thanks to their unique properties (namely their high affinity and selectivity in binding to a target molecule). In particular, aptamers can be used to obtain highly efficient and specific inhibitors of target proteins that can be applied in the design of new drugs. A number of aptamers are currently in different stages of clinical trials [8]. Macugen ( *Eyetech* *Pharmaceuticals* and *Pfizer* ), which is based on aptamer binding a human vascular endothelial growth factor (VEGF), has been certified as an efficient drug for the treatment of age-related macular degeneration [9, 10]. 

One of the most interesting and promising aspects in the field is designing aptamers that are capable of specific recognition of cells of a definite type through binding with certain dominants on their surface. In the review by B. Hicke  *et al.* [11], these compounds were referred to as *escort aptamers* . The use of escort aptamers as an addressing fragment opens wide possibilities for the targeted delivery of agents of different nature to cells of definite types. Today, a large number of escort aptamers directed toward various target cells have been obtained, and a wide range of applications for these aptamers for specific action on cells, diagnostics, and cell isolation have been described. The present review is devoted to the selection, design, and different aspects in the use of escort aptamers. 

## OBTAINMENT OF APTAMERS BY *in vitro* SElection 

**The general principle of the SELEX method**

**Table 1:** Comparison of the properties of NA aptamers and monoclonal antibodies

	Aptamers	Monoclonal antibodies
Selection method	*In vitro*selection	Hybridoma technology, including immunization of animals
Synthesis method	Chemical or enzymatic synthesis	Produced using cell cultures or laboratory animals
Limitations imposed on the target molecules	No limitations	Antibodies against non-immunogenic or toxic substances cannot be obtained
Affinity to the target	*K*_d_ ≈ 10^–10^–10^–7^ M	*K*_d_ ≈ 10^–10^–10^–7^ M
Specificity	High	High
Stability	Can re-naturate after heat treatment; long-term storage is possible	Irreversible denaturation after heat treatment; higher sensitivity to storing conditions
Immunogenicity	Not shown	High
Possibility of chemical modification	Wide	Limited

 DNA and RNA aptamers are obtained via *in vitro* selection from combinatorial libraries of nucleic acid molecules. A conventional library is a set of oligonucleotides with the randomized region consisting of 20–60 nucleotides flanked with the constant regions that are required for binding to primers and the PCR amplification of DNA. Currently, libraries containing both ssDNA and RNA molecules are widely used for the selection of aptamers. RNA aptamers are capable of forming a greater variety of spatial structures as compared with DNA aptamers, as a result of the presence of 2'-OH groups. However, RNA aptamers are more sensitive to the action of cell nucleases and require the introduction of additional protective groups [12]. 

The ssDNA libraries are obtained via the conventional methods for the chemical synthesis of oligodeoxyribonucleotides using a mixture of all four monomers when synthesizing a randomized fragment. In order to obtain an RNA library, the chemical synthesis of an ssDNA library containing the promoter sequence for T7 RNA polymerase at its 5'-terminal region is first performed. The ssDNA matrix is then used to obtain a dsDNA, which is subsequently applied for the synthesis of RNA via *in vitro* transcription. The general scheme of the SELEX method for DNA libraries comprises the following stages: incubation of a library with a target, separation of oligonucleotide–target complexes from the unbound oligonucleotides, disruption of the oligonucleotide–target complexes, and amplification of the bound molecules ( *Fig. 1A* ). For the selection of RNA aptamers, SELEX also comprises the following additional stages: production of the RNA library on the DNA matrix, reverse transcription of the bound RNA molecules to produce DNA, and DNA amplification ( *Fig. 1B* ). 

During the selection process, the library is enriched in sequences possessing increased affinity to the target. Five to fifteen rounds of selection are typically performed to obtain aptamers, depending on the values of the dissociation constant of the aptamer–target complex. After the dissociation constant ceases to decrease (i.e., the affinity of the library to the target stops rising), the enriched library is cloned, and the sequences of the individual aptamers are determined. The homology between the individual aptamers is then analyzed. On the basis of the results obtained, the aptamers are classified into several groups; their capability to interact with the target is assessed. The sequences with the maximum affinity to the target are selected for further studies. The secondary structure of aptamers is studied by analyzing their conserved motifs, by computer simulation, and chemical and enzymatic probing. The minimal size of an aptamer required for specific recognition of a target is determined at the next stage. For this purpose, a series of truncated variants of the aptamer is synthesized and the ability of these aptamers to bind to the target is determined.

**Fig. 2:**
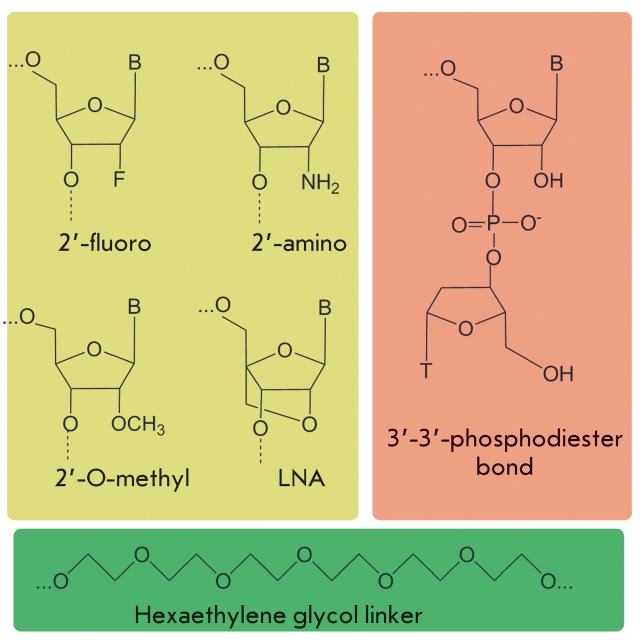
Chemical modifications of sugar-phosphate backbone increasing the resistance of escort aptamers in biological media.

Aptamers are usually characterized by high affinity to their targets. The characteristic values of the dissociation constant ( *K*_d_ ) for protein targets lie in the nanomolar and subnanomolar ranges (1 × 10 ^–10^ –1 × 10 ^–7^  М). In terms of their affinity and specificity, aptamers are similar to monoclonal antibodies; however, aptamers have a number of distinct characterisitcs ( *Table 1* ). Among these characteristics, the possibilities to produce an aptamer via chemical synthesis and to modify them chemically are the ones most worthy of note. 

**Chemical modifications of aptamers**

 The introduction of different modifications into escort aptamers enables one to considerably increase their stability in biological media, as well as their functionality. The introduction of different substituents at the 2' position of ribose ( *Fig. 2* ) (the most common modification used to increase an aptamer’s stability) helps to prevent the cleavage of aptamers by endonucleases. This type of modification is typically used to protect escort RNA aptamers, whereas escort DNA aptamers are more frequently used without any additional modifications. The major pathway of degradation of RNA aptamers in biological media is cleavage by pyrimidine endoribonucleases; therefore, as early as at the stage of construction of combinatorial RNA libraries for selecting escort aptamers, the pyrimidine nucleosides within them are substituted for their 2'-fluoro- and 2'-amino analogues by using the corresponding modified nucleoside triphosphates for synthesizing a library. It is possible to use T7 RNA polymerase [13] or its mutant version (capable of inserting these 2'-modified nucleoside triphosphates into RNA with higher efficiency) to integrate them into a growing RNA strand [14]. There is also mutant RNA polymerase inserting 2'-O-methyl analogues of nucleoside triphosphates [15, 16]; however, due to the problems associated with the reaction of reverse transcription of 2'-O-methyl-containing RNAs, the use of 2'-O-methyl-RNA libraries directly during the selection process has not yet become common practice [4]. 

In order to obtain 2'-O-methyl-containing aptamers, a quantity of ribonucleotides within a RNA aptamer are substituted for their 2'-O-methyl analogues, after the aptamers have been selected and their nucleotide sequences determined. The introduction of 2'-O,4'-C-methylene-linked bicyclic nucleotides (LNA – locked nucleic acids) is another way of increasing the stability of aptamers of a known sequence. This modification is used both in RNA [17] and DNA aptamers [18]. A number of nucleotides can be substituted for a flexible linker based on ethylene glycol for the purpose of minimizing the aptamer’s length and simultaneously increasing its resistance to endonucleases [18, 19]. Capping of the 3'-terminus with an additional thymidine residue linked via the 3'-3'-phosphodiester bond is used to prevent the cleavage of aptamers [17, 19].

Researchers either use the modified nucleoside triphosphates or modify the “ready-made” aptamers in order to introduce additional functional groups into an aptamer during the selection (see Review [4]). In the case of escort aptamers, the introduction of an aliphatic amino or sulfhydryl group to the 5'- or 3'-terminus of an aptamer is the most common. It allows one to synthesize the various conjugates of aptamers with toxins, antibiotics, fluorescent or photoreactive groups, nanoparticles, etc. (see Section Application of escort aptamers).

It must be kept in mind that the introduction of modifications into a “ready-made” aptamer may result in considerable changes in its molecular conformation, as well as a decrease in the aptamer’s affinity to its target. Therefore, in each case one must thoroughly select the type and position of these modifications. In addition, one must study the effect they have on the aptamer’s affinity to its target.

## OBTAINMENT OF ESCORT APTAMERS

**Fig. 3:**
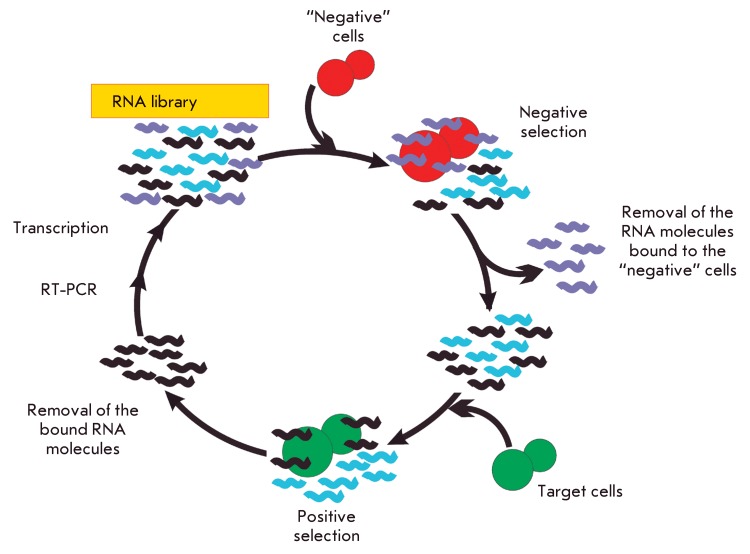
*.* The scheme of *in vitro* cell selection (including the negative selection stage) by the example of the RNA library.

When selecting escort aptamers *in vitro* , the individual proteins from the cell surface or whole cells are used as targets. The use of cells as targets has a number of advantages over using purified proteins: 

– No need for producing a pure protein to act as a target;

– The obtained aptamers possess high affinity to the target cells;

– The selection can be performed for the entire cell, even if a particular surface target protein is not known *a priori* ; and 

– The possibility to identify new, previously unknown specific markers on the cell surface emerges.

The protocol for cell selection of escort aptamers has specific features. The high number of surface dominants, which can either be unique for a definite cell type or be common to cells of various types, is one of the key problems that arise when using cells as targets. In order to eliminate the nonspecific aptamers binding to the molecular targets that are common to many cell types from the selection, an additional stage of counter-selection, or negative selection, is added to SELEX. Thus, to select aptamers that can bind to a certain protein on the cell surface, two cell lines are used: one of these cell lines (target cells) expresses the desired protein, whereas the second cell line (control “negative” cells) is represented by cells of the same type that do not express this protein. Sequential incubation of the nucleic acid library with the control cells and target cells enables one to select particular sequences that bind only to the desired protein on the cell surface .

The general scheme of cell selection of escort aptamers (by the example of an RNA library) is provided in *Fig. 3* . The initial oligonucleotide library is incubated with control cells, and the unbound molecules are isolated. They are then incubated with the target cells, and the unbound molecules are isolated again. After the cells have been disintegrated, the bound aptamers are extracted, amplified, and subsequently used in the next round of selection. 

**Fig. 4:**
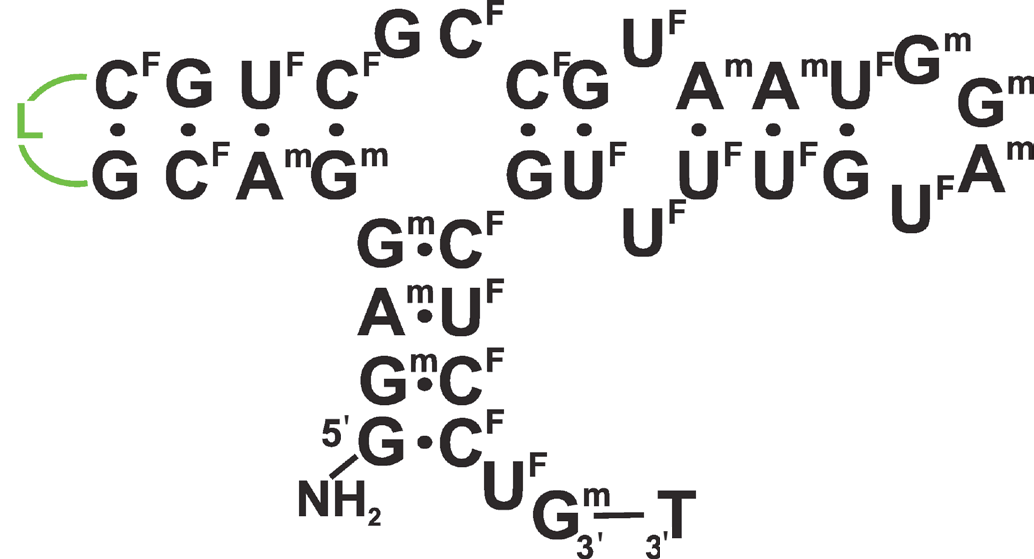
The proposed secondary structure of the modified ТТА1 aptamer binding to tenascin C [19]. Designations: N ^F^ – 2’-fluoro-2’-deoxyribonucleotide, N ^m^ – 2’-O-methylribonucleotide, NH _2_ – aminohexanol residue, L – hexaethylene glycol phosphate linker.

**Fig. 5:**
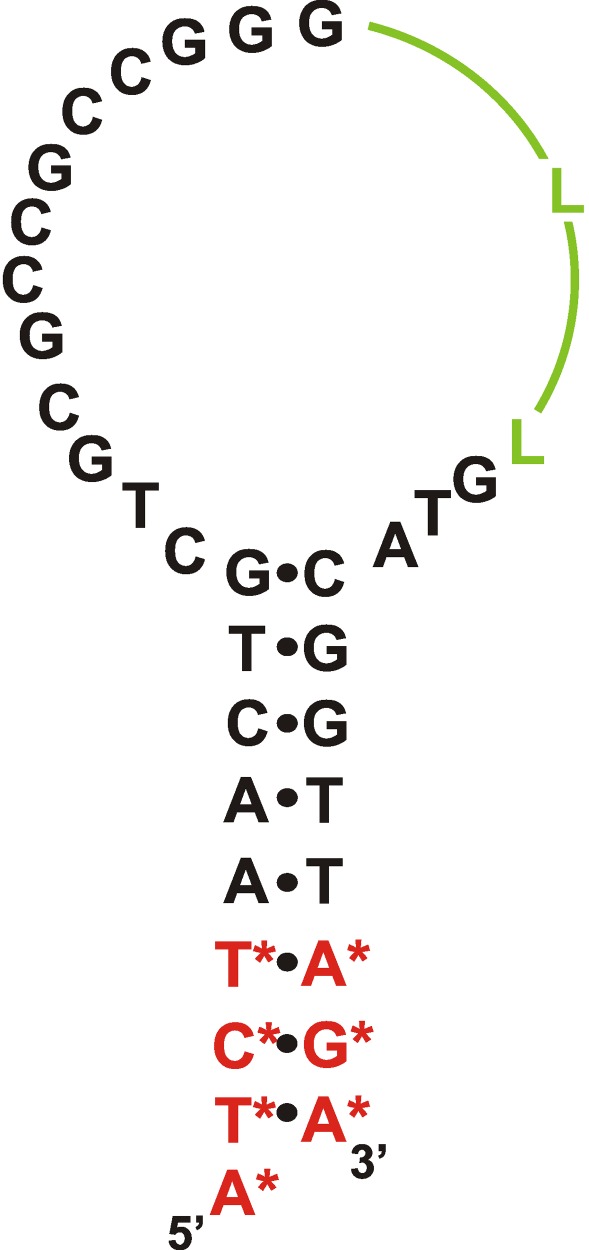
The proposed secondary structure of the modified C8FL aptamer against CCRF-CEM cells [18]. Designations: L – hexaethylene glycol phosphate, N ^*^  – LNA nucleotides.

The usage of cells as targets for *in vitro * selection was first described by K. Morris *et al* . [20]. DNA aptamers recognizing ghosts of human erythrocytes (haemoglobin-free cells that retain the same shape of the membrane as native erythrocytes) were obtained in that study. In order to produce aptamers, the ssDNA library was incubated with target cells. Then, the bound sequences were isolated via filtration through nitrocellulose filters. The resulting set of DNA molecules was amplified and used in the subsequent round of selection. After 25 selection rounds, two aptamer motifs comprised 25% of the total number of clones. Photoactivatable phenyl azide groups introduced into these two aptamers were used to demonstrate that they bind to the cell surface of various molecular targets. The study was the first example of using combinatorial libraries of nucleic acids for the selection of aptamers targeted at such complex objects as the cell membrane. 

**Aptamers recognizing malignant cells**

 The overwhelming majority of studies devoted to the selection of aptamers targeting living cells have focused on the search for sequences that can specifically bind to malignant cells. To this end, S. Lupold *et al* . [21] described the obtainment of 2'-F-modified RNA aptamers capable of binding to prostate-specific membrane antigen (PSMA). This protein is located on the cell membrane’s surface and is the marker of tumor cells of the prostate gland. The cells of healthy tissues are characterized by a very low level of PSMA, which considerably increases with the development of malignant tumors. A recombinant protein corresponding to the extracellular domain of PSMA, rather than whole tumor cells, was used as a target. After six rounds of selection, two RNA sequences ( **A9** and **A10** ) comprised 95% of the enriched library. A 56-nt aptamer **A10-3** was obtained via minimization of the length of the **A10** aptamer; an additional thymidine residue was linked to the 3'-terminus of ** A10-3** via a 3'-3'-phosphodiester bond for the purpose of protection from exonucleases. This aptamer was shown to specifically bind to PSMA-expressing LNCaP cells, and not to bind to PC-3 prostate cancer cells, which do not express this protein. 

C. Ferreira  *et al.* [22–24] also described the use of the fragments of individual surface proteins in studies devoted to the selection of DNA aptamers against the tumor marker surface glycoprotein mucin (MUC1). Mucin hyperexpression is typical of cancer cells. Immunogenic synthetic peptides (mucin fragments immobilized on a column with functionalized sepharose) were used as targets for the selection. After 10 rounds of selection, 12 aptameric sequences were obtained, one of which (aptamer **S1.3/S2.2** ) was capable of binding to mucin-producing tumor cells [23]. The same method was used to produce an additional four DNA aptamers, although recombinant mucin was used as a target [22]. The third variant selected against the mucin mimetic compound, O-glycosylated peptide, proved to be the most successful [24]. The DNA aptamer **5TRG2** , which was obtained via this method, is characterized by the highest affinity to the target peptide ( *K*_d_  = 18.6 nM) and is capable of not only selective binding to mucin on the cell surface, but also of penetrating into the cells via receptor-mediated endocytosis. 

It is noteworthy that the selection against whole cells is considered to be the most reliable and efficient method for aptamer production. Thus, D. Daniels  *et al* . [25] described the selection of DNA aptamers capable of binding to the surface of glioblastoma U251 cells. After 21 selection rounds, the resulting **GBI-10** aptamer, together with its homologues, comprised approximately 10% of the entire quantity of the selected sequences. It was ascertained via affinity purification of the cell extract using the **GBI-10 ** aptamer immobilized on magnetic particles that this aptamer is targeted against tenascin C (TN-C), the protein located mostly in the extracellular matrix. Hyperexpression of this protein is typical of a wide range of tumors. Selection was carried out at 4°C in order to prevent the penetration of aptamers inside the cells and to reduce the degree of aptamer degradation. The apparent dissociation constant of the aptamer–tenascin C complex was equal to 150 nM under the said conditions, whereas the *K*_d_ value increased by an order of magnitude as the temperature rose from 4 to 37°C. 

**Fig. 6:**
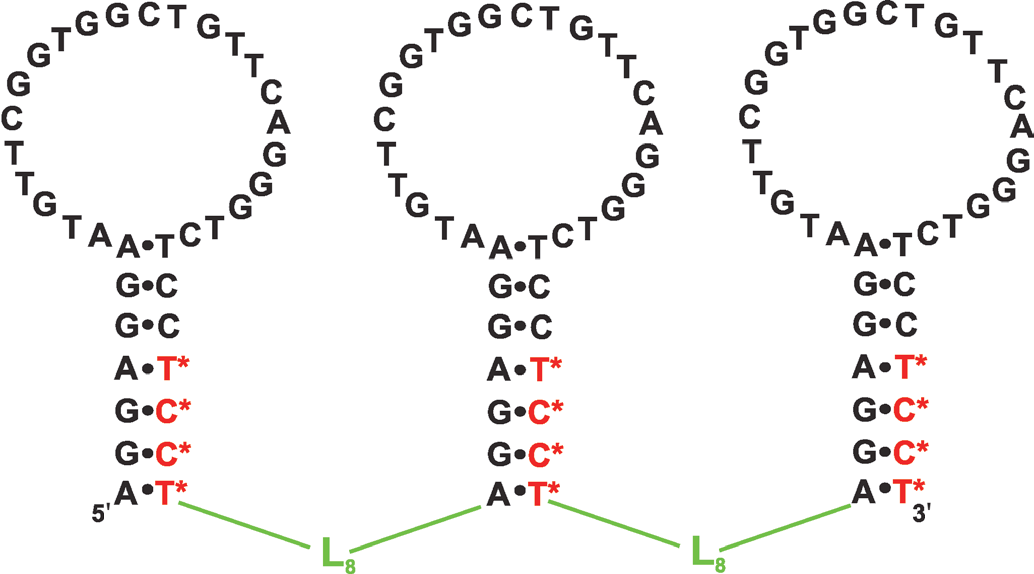
The proposed secondary structure of the trimer formed by the modified TD05.17 aptamer against Ramos cells [33]. Designations: L – hexaethylene glycol phosphate, N ^*^  – LNA nucleotides.

**Fig. 7:**
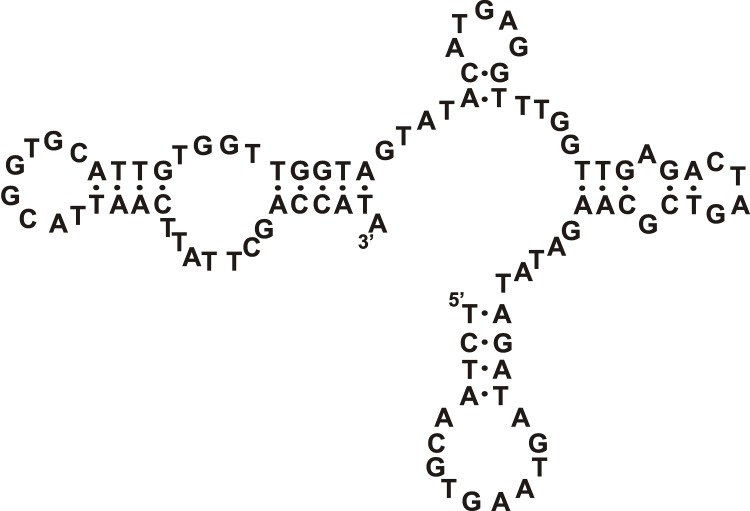
The proposed secondary structure of DNA aptamer III.1 against microvessels of rat brain tumor [37].

The considerably more stable complexes of 2'-F-RNA aptamers against tenascin C were obtained using three *in vitro* simultaneous selection protocols. In the first and second cases, a recombinant protein and glioblastoma U251 cells, respectively, were used as targets. In the third case, cross-selection was performed, which comprised two additional selection rounds with respect to the TN-C protein following the 9 rounds of selection with respect to glioblastoma cells [19]. All pyrimidine nucleosides within the RNA library were replaced by their 2'-F-analogues, in order to enhance their stability in biological media. The aptamers selected via all three methods had an appreciably high affinity to TN-C ( *K*_d_  = 1–10 nM). The aptamers obtained by selection against the individual protein and the aptamers obtained by selection against the cells contained similar sequences. The 55-nt aptamer  **TN-9** was truncated by 16 nucleotides; it also underwent several additional chemical modifications. Namely, several nucleotides were substituted for a hexaethylene glycol linker; most purine residues were substituted for their 2'-O-methyl analogues; the 3'-terminus was capped with a thymidine residue linked via a 3'-3'-phosphodiester bond; and amino groups were added to the 5'-terminus to produce bioconjugates. The resulting modified **ТТА1 ** aptamer ( *Fig. 4* ) retained a high level of affinity to the target protein ( *K*_d_  = 5 nM) and was characterized by high biological stability. 

W. Tan  *et al.* obtained DNA aptamers capable of binding to T-cell leukaemia CCRF-CEM cells [26]. Burkitt lymphoma B-cells (the so-called ‘Ramos cells’) were used as controls at the counter-selection stage. The resulting, highly affine aptamers were capable of not only selective binding to the CCRF-CEM target cells, but also of recognizing these cells in a mixture containing other cancer cell lines and cells from the medullary fluid of healthy individuals [27]. It turned out that the 88-nt **sgc8 ** aptamer with the highest affinity to the target cells ( *K*_d_  = 0.8 nM) binds to protein tyrosine kinase 7 (PTK7) on its surface [28]. PTK7 participates in signal transduction during the development and metastatic spread of malignant tumors. Moreover, the **sgc8 ** aptamer is capable of penetrating into CCRF-CEM cells, where it localizes in endosomes [29]. The **C8FL ** aptamer containing 33 nucleotides and a linker and possessing exceptional stability to serum nucleases and high affinity to target cells ( *K*_d_  = 1.53 nM) was obtained via minimization of the nucleotide sequence of the **sgc8** aptamer and the introduction of chemical modifications into its structure ( *Fig. 5* ) [18]. 

**Fig. 8:**
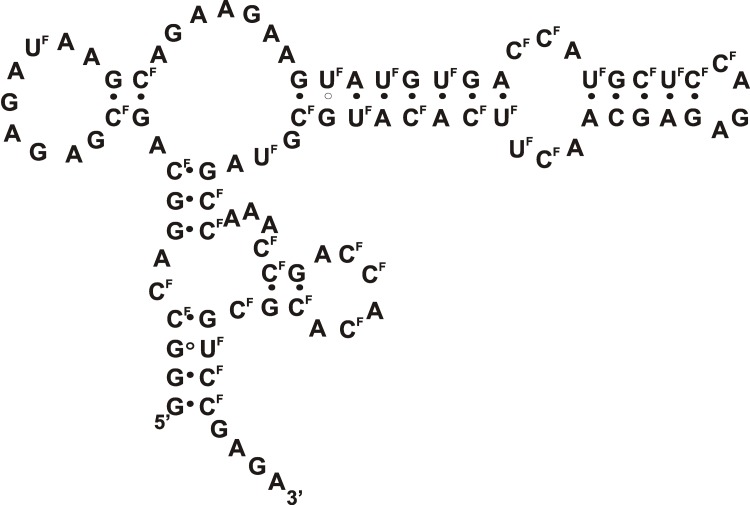
The proposed secondary structure of RNA aptamer А07 against the human transforming growth factor receptor [44]. Designations: N ^F^ – 2’-fluoro-2’-deoxyribonucleotide.

In 2009, A. Ellington  *et al.* [30] studied the specificity of the binding of a number of cell aptamers. In particular, they demonstrated that aptamers targeted at CCRF-CEM cells were capable of recognizing other types of malignant cells, which are typically capable of forming a monolayer. The authors assumed that the aptamers obtained in the studies conducted by W. Tan  *et al.* [26–29] could specifically recognize not a definite type of leukaemia cells, but cells capable of adhesion. The following arguments are given to support this assumption: 1) protein tyrosine kinase 7 participates in cell adhesion, 2) the CCRF-CEM cells capable of monolayer formation were used as targets during the selection, whilst the Ramos cells, which do not form a monolayer, were used as a negative control. In other words, it was possible to select aptamers against the cells capable of adhesion. However, it should be noted that it was the homologous **sga16 ** aptamer, and not the **sgc8 ** aptamer, that was used in the experiments described above. The affinity of the **sga16** aptamer to the target cells is lower than that of **sgc8 ** by an order of magnitude. Hence, one cannot unequivocally claim that all the aptamers obtained by W. Tan  *et al. * [26–29] are nonspecific to CCRF-CFM cells. 

W. Tan  *et al. * [31, 32] used Ramos cells not only as control cells, but also as selection targets; in the latter case, the SELEX cycle did not comprise the counter-selection stage [31]. It was demonstrated that the immunoglobulin M heavy chain bound to the membrane acted as a molecular target for aptamers on the surface of Ramos cells [32]. The tendencies of these aptamers to bind to the target cells only at low temperatures (the selection was carried out at 4°С) and their potential to bind not only to the cell surface of IgM, but also to the soluble IgM in blood plasma were their significant drawbacks. To solve this problem, the minimization of the structure of **TD05 ** aptamer (truncation of the nucleotide sequence from 48 to 37 nucleotides), combined with the substitution of four deoxyribonucleotides at the 3'-terminus for their LNA analogues, was performed [33]. The truncated modified aptamers **TD05.17 ** were then used to design tri- and tetrameric constructs in which the aptameric sequences were linked by non-nucleotide polyethylene glycol insertions (e.g., see *Fig. 6* ). The resulting multimers were specifically bound to Ramos cells at 37°С ( *K*_d_  = 256 nM for the trimer and 272 nM for the tetramer); they recognized heavy IgM chains and did not interact with soluble IgM. 

W. Tan  *et al* . also obtained DNA aptamers capable of distinguishing between two closely related acute myeloid leukaemia cell lines [34], between small-cell and nonsmall-cell lung cancer cells [35], and between hepatic cancer cells and normal hepatocytes [36]. 

M. Blank  *et al* . [37] obtained DNA aptamers which can selectively recognize brain tumor micro-vessels in rats and do not bind to healthy vessels. During the study, the DNA library was first incubated with the control cells represented by N9 microglial cells (brain monocytes), followed by incubation with the target cells represented by rat YPEN-1 immortalized endothelial cells transformed with the SV40 hybrid virus. Following the *in vitro* selection, the specificity of the binding of the target and control cells to each clone was determined and the histochemical staining of tumor vessels was performed. It turned out that the endothelial protein pigpen, whose synthesis increases in migrating and actively dividing endothelial cells, is the molecular target for the most active **III.1** aptamer ( *Fig. 7* ). The authors believe that this protein can be considered both as a new diagnostic angiogenesis marker and as a potential molecular target to block tumor angiogenesis. 

In 2011, E. Zueva  *et al.* published a study [38] devoted to the search for aptamers capable of distinguishing between highly mobile metastatic cells and malignant cells with low mobility. Two lines of transformed Syrian hamster fibroblasts (the control line with low mobility and the target cells with high mobility) were used to select 2'-F-containing RNA aptamers. Aptamers **Е10 ** and **Е37** were capable of binding to metastatic cells with high affinity and selectivity ( *K*_d _ values were 37 and 50 nM, respectively), and they were capable of suppressing cell migration at a concentration of 100 nM. The **E10** aptamer was also capable of suppressing cell invasion. 

J. Mi *et al.* [39] described the selection of aptamers against colorectal cancer cells with metastasis to the liver. It is noteworthy that model animals, rather than a cell culture, were used for the selection: a 2'-F-containing RNA library was intravenously injected into mice with a previously grafted liver tumor, followed by the extraction of the bound 2'-F-RNA from the liver. After it was intravenously injected to mice, the **14-16 ** aptamer was selectively bound to intraliver tumors. This aptamer was capable of penetrating into tumor cells and binding with helicase p68 in the nucleus and cytoplasm (hyperexpression of helicase p68 being typical of colorectal cancer). Thus far, this study is the only example of aptamer selection using multicellular organisms. 

**Aptamers binding to the surface receptors of cells**

 Cell receptors are considered to be an attractive therapeutic target. They can be neutralized by the action of aptamers blocking ligand-induced activation. Designing aptamers capable of specific blockade of certain receptors and the subsequent “switching-off” of the corresponding signal pathways provides the opportunity both to study the molecular mechanisms of their function and to investigate the diagnostics and therapy of different diseases. Several research groups have managed to obtain aptamers capable of binding to cell receptors. 

Recombinant proteins corresponding to the extracellular domains of receptors have been used as targets to obtain receptor-recognizing aptamers. The method was used to obtain 2'-fluoro-containing RNA aptamers capable of recognizing rat CD4 receptors [40], human CD4 receptors [41], and mouse CTLA-4 receptors [42]. C. Chen  *et al. * [43] described the production of aptamers against the mouse transferrin receptor. Fluorescently labelled RNA and DNA aptamers conjugated to streptavidin were used to demonstrate the ability of aptamers to penetrate into cells via endocytosis. Meanwhile, aptamer binding to the transferrin receptor had no effect on the interaction between this receptor and transferrin, since these aptamers can be used for the delivery of therapeutics without blocking the functions of the receptor necessary for the vital activity of the cells. 

Using the cell-SELEX method with a counter-selection stage, 2'-F-containing RNA aptamers against the human transforming growth factor β (TGF-β) receptor type III (TbRIII)(expressed on the surface of Chinese hamster ovary (CHO) cells) were selected [44]. In the study, parent CHO cells that do not express this protein were used at the negative selection stage. The **А07** aptamer ( *Fig. 8* ) that is capable of selective binding to the receptor TbRIII and forms with it a stable complex ( *K*_d_  = 2.47 nM) was obtained via selection. This aptamer was also shown to be capable of inhibiting association between the receptor TbRIII and its ligand, TGF-β _2_ . 

L. Cerchia  *et al.* [45] described the selection of 2'-F-RNA aptamers that bind to the mutant dimeric form of the human receptor tyrosine kinase RET ^C634Y^ , which is typically present upon multiple endocrine neoplasia type 2. During the selection, the library was incubated with cells expressing the mutant form of receptor RET ^C634Y^ (PC12/MEN2А). At the counter-selection stage, the oligonucleotide library was incubated with two types of control cells, the “parent” cells PC12 that did not express the target protein and the PC12/MEN2B cells that had a morphology similar to that of PC12/MEN2А but expressed the monomeric form of receptor tyrosine kinase RET ^М918Т^ . The **D4** and **D24** aptamers obtained after 15 selection rounds were capable of efficiently binding to the target cells ( *K*_d_  = 40 nM) and suppressing the activity of RET by 70% at a concentration of 200 nM. It should be noted that the **D4** aptamer was also capable of binding to PC12/MEN2B cells, although the degree of binding was considerably lower (by a factor of approximately 2.5 as compared with that of the target cells PC12/MEN2А). 

Unfortunately, the use of cross-selection to obtain aptamers against the receptor RET ^C634Y ^ (seven rounds of selection for PC12/MEN2А cells and four rounds of selection for the purified recombinant protein [46]) failed to yield better results. The obtained **E38** aptamer had a considerably different structure in comparison with that of the aptamers against the target cells and was characterized by lower affinity to PC12/MEN2А cells ( *К*_d_  = 100 nM). It was also incapable of inhibiting the activity of the receptor tyrosine kinase. 

**Aptamers capable of recognizing undifferentiated cells**

 The production of aptamers that can bind to undifferentiated cells is challenging. C. Wang * et al. * [47] selected DNA aptamers capable of distinguishing between differentiated PC12 cells and the undifferentiated “parent” cells that were used as the control cells at the counter-selection stage. Six selection rounds were sufficient to obtain aptamers that were capable of recognizing the differentiated cells and did not bind to the undifferentiated cells. Aptamers against undifferentiated cells types, e.g., against stem cells, can be used for cell isolation and purification in regenerative medicine, which is a rapidly developing field of medicine. The studies conducted by K. Guo  *et al. * [48, 49] were devoted to the creation of DNA aptamers for cell isolation and immobilization. In particular, DNA aptamers capable of binding to mature mesenchymal stem cells were obtained [49]. After 12 selection rounds without counter-selection, aptamers capable of selectively recognizing the target cells, among other medullary cells, were obtained; the possibility of using them to isolate stem cells from bone marrow was demonstrated. J. Hoffmann *et al.* [50] produced DNA aptamers capable of binding to the precursors of porcine endothelial cells, which were subsequently used to immobilize the cells on the surface of polytetrafluoroethylene or polydimethylsiloxane disks (see Section Application of escort aptamers). 

**Identification of new biomarkers via the cell selection of aptamers**

**Fig. 9:**
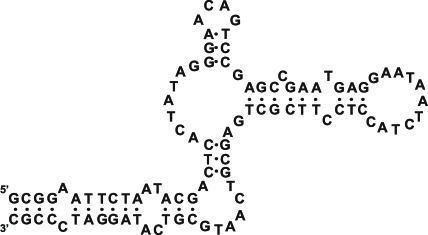
The proposed secondary structure of DNA aptamer ZE2 against the hepatitis C virus coat protein [70].

 The *in vitro* selection of aptamers using living cells enables to identify new biomarkers typical for cells of a definite type, after the aptamers have been selected and the surface proteins binding to these aptamers have been revealed. Existing methods for searching for biomarkers (Western blotting, screening of mRNAs using quantitative PCR or chips, 2D electrophoresis coupled with mass spectroscopy) are not sufficiently efficienct; their common drawback being the possibility of false positive and false negative results, which often occurs. The general strategy for the search for biomarkers using aptamers (AptaBiD, Aptamer-facilitated Biomarker Discovery) formulated by M.V. Berezovski *et al* . [51] enables to overcome these difficulties. The probability of obtaining false positive results decreases through multiple selection rounds, which eliminate the impact of such random factors as the stochastic differences between cells of the same type and unintended variations at all stages of cell treatment. Meanwhile, the exponential enrichment of the library during the selection allows to reveal even insignificant distinctions between the control cells and the target cells, if they are retained from round to round. This reduces the probability of obtaining false negative results. To confirm the high potential of the AptaBiD strategy, it was used to search for biomarkers determining the differences between mature and immature dendritic cells. As a result, both previously known biomarkers of dendritic cells and six new biomarkers of immature dendritic cells were revealed. A significant feature of this method worth noting is that it does not involve the cloning and sequencing stages; the enriched libraries, rather than individual aptamers, are used for the search for biomarkers. Thus, the process becomes both quicker and cheaper. 

**Aptamers recognizing the surface proteins of microorganisms**

 In addition to cultured cells, pathogenic microorganisms can also be used as targets for cell selection. The aptamers obtained via this method can be subsequently used in the diagnostics and therapy of infectious diseases, as well as for the quantitative determination of microorganisms. 

**Fig. 10:**
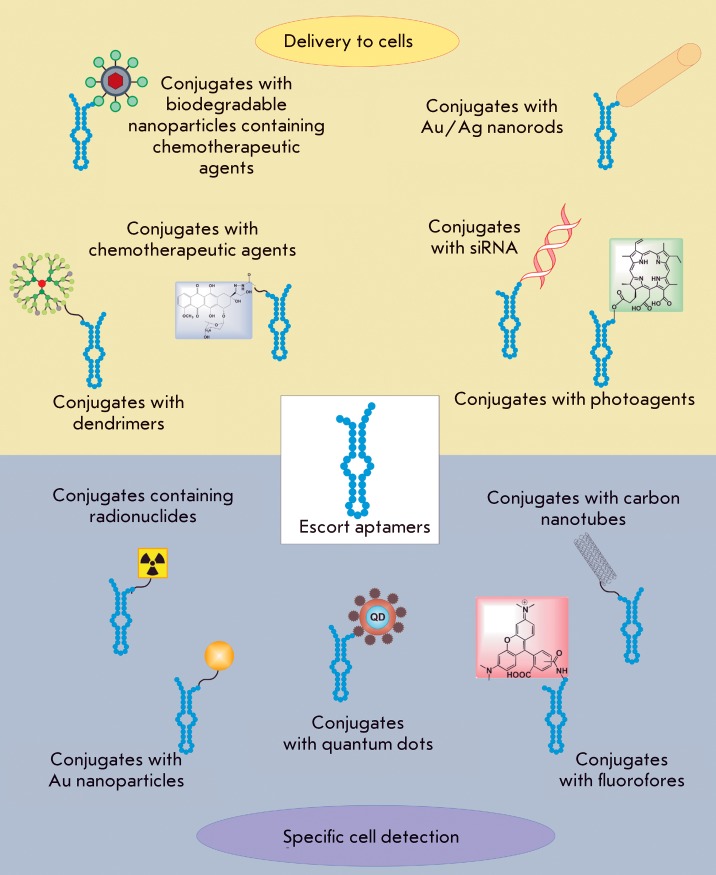
The main types of conjugates of escort aptamers used for targeted cell delivery and specific cell detection.

**Table 2:** Escort aptamers and their use for the delivery of various therapeutics into cells and for specific cell detection

Aptamer	Target	Selection method	Application
2'-F-RNA	Prostate-specific membrane antigen (PSMA)	Selection against recombinant protein – extracellular domain of PSMA [21]	Delivering gelonin [71] and doxorubicin [72] into LNCaP tumor cells.Delivering siRNA into LNCaP cells [74–77].Delivering nanoparticles containing antitumor therapeutic docetaxel into LNCaP cells [82–84].Imaging of LNCaP cells using aptamer-conjugated gold nanoparticles [92] or aptamer-conjugated luminescent CdSe–CdTe crystals [90].Electrochemical detection of prostate cancer cells using an aptamer immobilized on an Au-electrode [91].Detection of PSMA on LNCaP cell surface via proximity ligation assay [96]
DNA	Protein tyrosine kinase 7 (PTK7)	Selection against the CCRF-CEM cells (the precursors of T-cell acute myeloid leukaemia cells) [26]	Delivering doxorubicin [73], Au/Ag nanorods [80, 81], and poly(amidoamine)-based dendrimers [87] into CCRG-CEM cells .Investigation of the distribution of PTK7 receptors over the cell surface using aptamer–fluorescein conjugates [93].Reversible fluorescent labelling of CCRF-CEM cells using aptamer-conjugated quantum dots Qdot525 [94]
2'-F-RNA	Rat CD4 receptor	Selection against recombinant protein [40]	Delivering siRNA into cells [78]
2'-F-RNA	HIV-1 coat protein	Selection against recombinant protein gp120 [79]	Delivering siRNA into HIV-1-infected cells [7, 79]
DNA	Mucin (MUC1)	Selection against synthetic peptides (mucin fragments) [23, 24]	Photodynamic therapy using photoreactive conjugates of the aptamer and chlorine e6 [24].Tumor imaging using radioactive isotopes (^99^Tc) [88]
DNA, RNA	Mouse transferrin receptor (TfR)	Selection against recombinant protein – extracellular domain of TfR	Delivering lysosomal enzymes into cells to treat lysosomal storage diseases [43]
2'-F-RNA	Tenascin C	Cross-selection using a recombinant protein and U251 cells [19]	Tumor imaging using radioactive isotopes (^99^Tc) [89]
DNA	Membrane-bound IgM heavy chain	Selection against Burkitt lymphoma B-cells (Ramos cells) [31]	Micelles for delivering various pharmaceutics into cells [86].Test strips based on TD05 and TE02 aptamers for the detection of Ramos cells in blood samples [95]
DNA	*B. thuringiensis*bacteria	Selection against*B. thuringiensis *spores	Detection of*B. thuringiensis *spores using aptamer-conjugated CdSe-ZnS quantum dots [61]
RNA	*E. coli*bacteria	Selection against*E. coli *DH5α strain [67]	Potentiometric detection of*E. coli *using aptamers-conjugated carbon nanotubes [67].Detection of*E. coli*via quantitative RT-PCR of RNA aptamers bound to the bacteria [98]
RNA	*S. typhi*bacteria	Selection against the major protein of*S. typhi*microvilli [63]	Potentiometric detection of*S. typhi *using aptamer-conjugated carbon nanotubes [97]
DNA	Mesenchymal stem cells	Selection against porcine mesenchymal stem cells [49]	Isolation of stem cells from bone marrow using aptamers immobilized on magnetic particles; cell sorting using fluorescent aptamer conjugates [49]
DNA	Precursors of porcine endothelial cells	Selection against CD31-positive cells from porcine blood [50]	Immobilization, growth, and differentiation of the precursors of endothelial cells on the surface of disks with immobilized aptamers (a model of vascular implants) [50]

M. Homann and H. Göringer [52] obtained RNA aptamers capable of binding to the living trypanosomes *Trypanosoma brucei* , parasitic protozoans causing the sleeping sickness. Two *Tr. brucei* strains were used as targets for the selection. The RNA library was incubated with parasitic organisms that were present at the bloodstream stage; the unbound molecules were removed via centrifugation. The resulting aptamers were capable of binding to the organisms of both strains at the bloodstream stage ( *K*_d_  = 60 nM), whilst being incapable of recognizing *Tr. brucei* at other stages of development. The methods of photoaffinity modification and fluorescent microscopy with the fluorescent-labelled aptamer **2-16** were used to ascertain that the protein with a molecular weight of 42 kDa located in the trypanosome flagellar pocket acts as a target for this aptamer. After binding to this protein, the aptamer penetrates into the trypanosome via receptor-mediated endocytosis and is subsequently located in endosomes. As shown by the example of the **2-16**  aptamer conjugated with biotin, these aptamers can be used to deliver other substances into trypanosomes [53]. Pyrimidine nucleosides were replaced by their 2'-amino or 2'-fluoro analogues in order to increase the stability of the **2-16**  aptamer in biological media. As a result of the modification, the aptamer containing 2'-NH _2_ groups lost its ability to bind to trypanosomes; in contrast, the 2'-F-modified aptamer retained its affinity to these organisms ( *K*_d_  = 70 nM) and was characterized by high resistance to serum nucleases [54]. The use of modified RNA libraries containing 2'-fluoro or 2'-amino pyrimidine nucleotides during the selection was more successful. Living trypanosomes were used as targets when selecting 2'-amino-containing RNA aptamers. The resulting aptamer possessed affinity to trypanosomes, being virtually equal to that of the **2-16 ** aptamer ( *K*_d_  = 70 nM), and bound to these organisms within the limited area around the flagellum [55]. Selection against the purified surface protein sVSG was used to obtain 2'-F-containing RNA aptamers capable of binding to the entire trypanosome surface [56, 57]. 

**Fig. 11:**
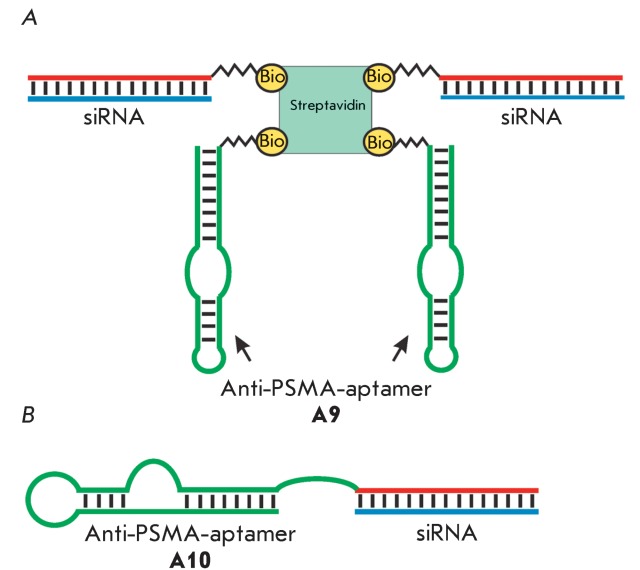
Schematic representation of chimeric constructs for siRNA delivery into PSMA-positive cells. A. The conjugate of biotinylated anti-PSMA aptamer and siRNA connected via streptavidin [74]. B. Chimeric RNA built from an anti-PSMA aptamer and siRNA [75]. Bio – biotin residue.

2'-F-RNA aptamers against another type of trypanosomes ( *Tr. crusi* , the agent of Chagas disease) were also obtained [58]. At the trypomastigote stage, *Tr. cruzi * bind to the host cells and penetrate into them through interaction with the extracellular matrix proteins of the host cells. 2'-F-pyrimidine-containing RNA aptamers at a concentration of 1 µM blocked the penetration of these parasitic organisms into the cells by 50–80%. 

 Aptamers capable of binding to different types of bacteria generate significant interest. Thus, the selection of DNA aptamers with respect to the causative agent of tuberculosis *Mycobacterium tuberculosis* was performed [59]. A single introduction of 0.8 µg of the resulting **NK2 ** aptamer resulted in a decrease in the numbers of microbacteria in tuberculosis-infected mice, alleviated disease presentations, and also increased the lifetime [59]. The potential use of the **NK2**  aptamer in tuberculosis therapy is assumed. Aptamers capable of specific binding to the spore surface of the causative agent of anthrax * Bacillus anthracis* [60] and crystal-forming bacteria *B. thuringiensis* [61], salmonellae *Salmonella enterica * [62] and *S. typhi* [63], staphylococci *Staphylococcus aureus * [64], lactic acid bacteria *Lactobacillus acidophilus* [65], *Escherichia coli* [66, 67], and *Campylobacter jejuni* bacteria [68] have also been obtained. 

 Escort aptamers capable of binding to “foreign” proteins located on the surface of infected cells should be considered separately. A. Barfod *et al.* [69] obtained aptamers against the PfEMP1 protein, which is expressed on the surface of erythrocytes infected with the malaria parasite *Plasmodium falciparum* . This protein facilitates erythrocyte aggregation (formation of the so-called ‘rosettes’) and adhesion of the infected erythrocytes to the walls of minute blood vessels. The recombinant DBL1α protein (the semi-conserved N-terminal domain of the PfEMP1 protein responsible for rosettes formation) was used as a target for the selection of 2'-F-pyrimidine-containing RNA aptamers. The resulting aptamers at a concentration of 387 nM (12 µg/ml) caused the almost complete disintegration of rosettes in a cell culture, which allows one to view them as potential anti-malaria agents. 

2'-F-RNA aptamers against gp120, the human immunodeficiency virus type 1 (HIV-1) coat protein, were obtained by selection with respect to the recombinant protein; these aptamers were capable of binding to gp120 on the surface of the infected cells (see Review [7]).

F. Chen  *et al.*  [70] described a procedure for obtaining DNA aptamers against the E2 protein expressed on the cell surface (the hepatitis C virus coat protein). The same cell line incapable of expressing this protein was used for counter-selection. Among the resulting series of aptamers, the **ZE2 ** aptamer possessed the highest affinity to the surface protein ( *K*_d_  = 1 nM) ( *Fig. 9* ). At a concentration of 100 nM, this aptamer was both capable of binding to viral particles, as well as blocking their fusion with cells. These results enable one to assume that the **ZE2** aptamer can potentially be used both for the diagnostics of hepatitis C and for the treatment of patients suffering from this disease, as well as for studying the virus-cell interactions. 

The following section describes the design of systems centred on the cell delivery of therapeutic agents, diagnosing various diseases, and determining the pathogenic microorganisms in the environment and food products based on escort aptamers.

## Application of escort aptamers

Numerous multifunctional constructs have been designed on the basis of escort aptamers, where an aptamer acts as a directing component ensuring the specific recognition of cells or target tissues. The aptamers that are capable of penetrating into cells via receptor-mediated endocytosis after they bind to the surface proteins can be used as a platform to design highly specific therapeutic agents that can impose targeted action on cells of a definite type. Another actively developing direction in the application of escort aptamers is their use in the design of a highly precise diagnostic system enabling the detection of target cells, among other cells, in the organism. The schematic presentation of the major types of conjugated escort aptamers that are currently being used to deliver therapeutic agents to cells and perform specific cell detection is provided in *Fig. 10* . The data on the application of escort aptamers are briefly summarized in *Table 2* . 

**The use of escort aptamers to deliver various therapeutic agents to cells**

 The anti-PSMA-aptamers **A9** and **A10** , which bind to the prostate-specific membrane antigen, are among the most popular candidates for the design of a delivery systems [20]. PSMA is capable of penetrating into cells via clathrin-mediated internalization [97]; hyperexpression of this protein is a characteristic feature of many tumors. It was a combination of these factors that led to the significant interest pertaining to the use of anti-PSMA-aptamers as an addressing fragment in the delivery of various anti-tumor agents into cells. Thus, the conjugation of anti-PSMA-aptamer A9 with the protein toxin gelonin resulted in a ~180-fold increase in cytotoxicity with respect to the target cells, in comparison with that for unbound gelonin (IC _50_  = 27 nM for the conjugate and 5 µM for gelonin) [71]. Meanwhile, in the case of control cells incapable of expressing the PSMA protein, the cytotoxicity of the aptamer-gelonin conjugate was lower than that of the unbound gelonin (IC _50_  = 15 µM), which attests to the fact that this conjugate is highly selective. V. Bagalkot  *et al.*  [72] used the **A10** anti-PSMA-aptamer to deliver doxorubicin into cells. The anthracycline antibiotic agent doxorubicin is used for the therapy of a wide range of diseases, such as leukaemia, malignant lymphomas, sarcomas, and cancers of various etiologies. However, the drawbacks include toxic side effects, in particular, cardiotoxicity. The ability of doxorubicin to intercalate between the base pairs of double-stranded nucleic acids was used to obtain the conjugates [72]. The intercalation of doxorubicin into a double-stranded fragment of the **A10** aptamer yielded non-covalent conjugates. The cytotoxicity of these conjugates with respect to LNCaP target cells was comparable to that of unbound doxorubicin of the same concentration (IC _50_  = 5 µM). The cytotoxicity of conjugates with respect to PC3 control cells was significantly lower. The **sgc8с ** escort aptamer (“truncated” variant of the **sgc8** aptamer) was used to deliver doxorubicin into human leukemic lymphoblasts (CCRF-CEM cells). Y. Huang *et al. * [73] obtained the doxorubicin– **sgc8c ** aptamer conjugate, with doxorubicin bound to the 5'-terminus of the aptamer via an acid-labile hydrazone bond that was hydrolyzed after the conjugate had penetrated into the cell. It has been shown that these conjugates can selectively penetrate into CCRF-CEM cells via receptor-mediated endocytosis; their cytotoxicity with respect to CCRF-CEM cells being comparable to that of unbound doxorubicin (IC _50_  = 0.3 µM). As opposed to unbound doxorubicin, the aptamer-doxorubicin conjugate showed no toxicity with respect to the control Ramos cells. Thus, binding of escort aptamers to chemotherapeutic agents makes it possible to reduce their toxic effects on tumor cells only. It can be anticipated that these conjugates will be used to design novel agents for anti-tumor chemotherapy with minimum adverse effects. 

Escort aptamers were also used to deliver small interfering RNA (siRNA) into cells. Several types of constructs to deliver siRNA were designed on the basis of anti-PSMA-aptamers. The tetrameric biotin-streptavidin complex containing two biotinylated strands of the **A9 ** aptamer and two biotinylated siRNA molecules targeted against mRNAs of the lamin A/C or GADPH genes ( *Fig. 11A* ) was used to deliver siRNAs into PSMA-positive tumor cells [74]. These complexes could penetrate into cells without the use of transfectants. At a concentration of 22.5 nM, they suppressed target gene expression by 50–80%; the efficiency of the suppression was identical to that of the corresponding siRNAs delivered into cells using Oligofectamine. The chimeric constructs were designed which consisted of a joint nucleotide sequence containing the **A10** aptamer and one of the siRNA strands with the complementary second siRNA strand ( *Fig. 11B* ) [75]. At a concentration of 400 nM, these constructs can penetrate into PSMA-positive cells without transfectants and almost completely suppress the expression of the *bcl2* and  *plk1* target genes. With the aim of optimizing the structure of chimeric constructs, the targeting aptamer was truncated from 71 down to 39 nucleotides, which simplified the chemical synthesis of both components of the construct. A number of modifications increasing the specificity and efficiency of the interaction with the mRNA target were introduced into the siRNA structure. A polyethylene glycol residue with a molecular weight of 20 kDa was bound to the siRNA passenger strand, which increased the half-circulation time of the chimeric constructs in mouse blood from 35 min to 30 h. The obtained preparation resulted in a considerable regression of the PSMA-positive tumor in mice after the injection of five 0.25 nmol doses [76]. 

**Fig. 12:**
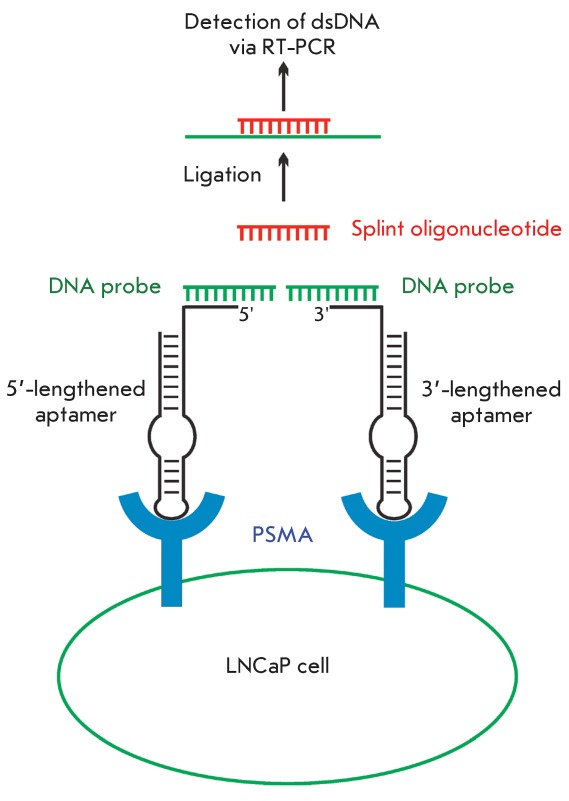
Detection of prostate-specific membrane antigen using anti-PSMA aptamers by the proximity ligation assay [96]. The method includes the following stages: 1) formation of the complementary complexes of aptamers with DNA probes; 2) binding of aptamers to the adjacent sites on the cell surface, thus positioning DNA probes closer to each other; 3) hybridization of both DNA probes with the splint oligonucleotide; 4) ligation of DNA probes in the resulting complex; 5) detection of dsDNA via real-time PCR.

Another variant of a chimeric construct containing two molecules of anti-PSMA-aptamer **A10-3 ** was described in [77]. In this construct, one of the siRNA strands targeted against mRNA of the eukaryotic elongation factor 2 (eEF2) is inserted between two aptameric sequences, the second strand being complementary [77]. At a concentration of 2 µM, these conjugates caused suppression of the growth of the target cells by 95%, with no impact on the growth of PSMA-negative control cells. 

An interesting construct containing the “addressing” aptamer and siRNAs was described in [78]. Phage φ29 RNA capable of multimeric complex formation via interaction between RNA loops was used to design these constructs. A phage RNA fragment was bound to each component of the construct, i.e., to the aptamer recognizing the CD4 receptor [40], to siRNAs targeted against the mRNAs of various apoptotic factors, and to the fluorescent dye. At a concentration of 100 nM, the resulting trimers containing an aptamer, siRNA, and a reporter group were able to penetrate into the CD4-positive cells and inhibit the expression of the target genes.

Chimeric constructs [7, 79] designed according to the principle proposed by J. McNamara  *et al.*  [75] and consisting of a 2'-F-RNA-aptamer recognizing the viral protein gp120 on the cell surface and siRNA targeted against the *Тat/Rev* of HIV-1 RNA were used to act upon HIV-1 infected cells. These constructs (at a concentration of 400 nM) have the ability to inhibit HIV-1 replication in a cell culture [79]. The use of these constructs to suppress HIV-1 replication in mice has also been reported [7]. 

The **sgc8c** aptamer conjugated with Au-Ag nanorods was used to impose photothermal action on leukaemia cells [80, 81]. The nanorods are heated to 50°C under laser irradiation, which results in cell death through thermal shock. Aptamers conjugated with nanorods (approximately 80 aptameric molecules were bound to a nanorod) were capable of selective penetration into the target cells; approximately 90% of the cells died after exposure to radiation [81]. 

 Reactive agents for photodynamic therapy (aptamers covalently conjugated to chlorine e6) have been designed on the basis of aptamers capable of binding to the surface protein mucin [24]. These conjugates can selectively penetrate into mucin-expressing tumor cells and cause their death after exposure to radiation; the efficiency of the conjugates was 500-fold higher than that of unbound chlorine. Meanwhile, the conjugates showed no toxicity towards healthy cells.

The DNA aptamer against mouse transferrin receptor was used to deliver the lysosomal enzyme α- *L* -iduronidase into cells [43]. It was demonstrated that the aptamer-enzyme conjugates penetrated into mouse fibroblasts deficient in this enzyme and were further transported to lysosomes, where the introduced enzyme was capable of both functioning and facilitating the recovery of the cell metabolism. The results obtained make aptamers against the transferrin receptor conjugated with lysosomal enzymes promising therapeutic agents for the treatment of diseases associated with the lysosomal function disorder. 

Various carriers bound to the addressing aptamers are also used to ensure specific delivery of therapeutic agents. Thus, nanoparticles composed of the copolymer of lactic acid and glycolic acid (PLGA) with encapsulated docetaxel were covalently bound to the molecule of anti-PSMA-aptamer **A10** [82–84]. The resulting conjugate was capable of specific binding to LNCaP cells expressing the PSMA protein and penetrating into them [82, 85]. Mice with a grafted prostate tumor were used to demonstrate that anti-PSMA-aptamers conjugated with nanoparticles based on PLGA containing docetaxel can efficiently suppress tumor growth and can even result in complete remission [83]. 

The construction of micelles based on the **TD05** aptamer with the stearic acid bound to it was described in [86]. These micelles were characterized by increased affinity to the target cells in comparison with that of the individual **TD05** aptamer, and they were capable of specific penetration into the cells. It is not anticipated that these micelles will be used further to deliver therapeutics into cells. 

J. Zhou  *et al.* [87] proposed using polyamidoamine (PAMAM) dendrimers as carriers for therapeutic delivery [87]. **Sgc8c** aptamer-conjugated dendrimers proved capable of selective and efficient binding to CCRF-CEM cells and penetrating into them. The size of the aptamer-dendrimer conjugate is approximately 8 nm, the optimal size for using these conjugates as a platform for the delivery of therapeutic agents. 

Thus, the use of aptamers for targeted delivery of nanoparticles with anti-tumor agents into tumor tissues is a promising avenue in the development of novel anti-tumor therapeutic strategies.

**The use of escort aptamers for specific cell detection**

 The capability of escort aptamers to selectively recognize cells of a definite type enables one to use them to design highly specific detection systems. The introduction of different types of labels into aptamers allows one to use them for cell detection in cultures, biological samples, and in living multi-cellular organisms. Thus, radio-labeled aptamers have been used for tumor imaging in mice. Conjugates of the anti-tenascin aptamer **TTA1** and anti-mucin aptamers with chelating agents capable of binding to ^99^ Tc were used for the imaging of glioblastoma and breast cancer xenografts in mice [88, 89]. 

Various systems for detecting prostate cancer have been designed on the basis of anti-PSMA-aptamers. T. Chu  *et al.* [90] obtained the anti-PSMA-aptamer **A9** conjugated with luminescent CdSe/CdTe crystals (the so-called quantum dots) for the imaging of PSMA-positive cells. These conjugates could bind to LNCaP cells distributed over the model tissue (3D collagen matrix) with high levels of efficiency and specificity. “Double” constructs consisting of the anti-PSMA-RNA-aptamer **A10** and the peptide aptamer against PSMA-negative cells immobilized on a gold electrode were used for the electrochemical detection of two types of prostate cancer cells: those that contained PSMA on their surface (PSMA-positive) and those that did not contain the protein (PSMA-negative) [91]. D. Javier  *et al. * [92] introduced additional oligonucleotide fragments, which were complementary to 24-membered oligonucleotides covalently bound to gold nanoparticles, into anti-PSMA-aptamers in order to perform the imaging of PSMA-positive cells. The binding of these complexes to PSMA on the cell surface was observed by reflected light detection using a confocal microscope. 

DNA-aptamer  **sgc8** conjugated to fluorescein was used to study the distribution of the receptors of PTK7 over the cell surface via fluorescence correlation microscopy [93]. A new method for the reversible fluorescent labelling of live cells was proposed in [94] and was illustrated by the example of CCRF-CEM cells. Aptamer  **sgc8 ** conjugated with the quantum dot Qdot525 selectively bound to cells; after treatment with DNAse, the conjugates were completely removed from the cell surface, whereas the cells retained their viability. Fluorescence-activated cell sorting, enabling the rapid and efficient isolation of cells of a definite type, is a promising application of this method. Fluorescence-activated sorting of stem cells using aptamer–fluorescein conjugates was also described by K. Guo  *et al.* [49] 

Aptamers **TD05** and **TE02 ** targeted against Ramos lymphoma cells were used when designing biosensor test strips for the rapid determination of malignant cells circulating in the blood stream [95]. The test zone of the biosensor contains the **TD05** aptamer conjugated with gold nanoparticles. These conjugates form coloured complexes with Ramos cells, which subsequently migrate along the strip until they are captured in the indicator zone of the biosensor through the binding of Ramos cells to the immobilized **TE02**  aptamer. As a result, a characteristic red band is generated. These biosensors can be used for cell detection directly in blood samples; a visual qualitative assessment or quantitative determination can be performed using a portable scanner. 

The use of a so-called ‘proximity ligation assay’ was proposed as means to detect the PSMA protein on the cell surface ( *Fig. 12* ) [96]. The system is composed of **A9** aptamers containing additional oligonucleotide fragments on their 3'- or 5'-termini, DNA probes that are complementary to each fragment, and the splint oligodeoxyribonucleotide, which is partially complementary to each DNA probe. Since PSMA is a dimer, when aptamers bind to PSMA on the cell surface, DNA probes become sufficiently close that they can be bound by a splint oligomer via the formation of a complementary complex. The DNA probes within this complex are then ligated; subsequently, real-time PCR is used to detect the resulting dsDNA. This method is highly sensitive and enables the detection of 10 LNCaP cells (prostate cancer) in the presence of 10 ^5^ HeLa cells that do not contain the PSMA protein on their surface. 

It was recently suggested that the aptamers binding to bacteria can be used for the design of biosensors for use in the detection of pathogenic microorganisms. DNA aptamers with bound CdSe-ZnS quantum dots were used for the fluorescent detection of *B. thuringiensis* [61]. RNA aptamers immobilized on the surface of single-walled carbon nanotubes were used to design potentiometric biosensors to determine *E. coli * [67] and *S. typhi* [97] and to demonstrate that they can be used for selective detection of these bacteria. A method based on the quantitative RT-PCR of RNA aptamers bound to bacteria immobilized on magnetic particles was developed for the detection of *E. coli* [98]. Novel aptamer-based biosensors could be used for highly selective determination of bacteria in the environment and food products and for diagnosing infectious diseases. 

Another promising direction in the use of escort aptamers is the selective isolation of cells and cell immobilization. Thus, a system for the selective isolation of stem cells from bone marrow was designed on the basis of magnetic particles conjugated with aptamers capable of binding to porcine stem cells [49]. DNA aptamers capable of binding to the precursors of porcine endothelial cells were immobilized on the surface of polytetrafluoroethylene or polydimethylsiloxane disks [50]. It turned out that aptamer-coated disks can be used to selectively isolate endothelial precursor cells, which can be subsequently differentiated into vascular endothelial cells while remaining bound to the disks. The assumption is that this approach could be used for the epithelialization of vascular implants, thus reducing the risk of implant rejection.

## CONCLUSIONS

A number of escort aptamers capable of specific and efficient binding to cells of a definite type were recently obtained and approaches have been developed aimed at enhancing the stability of escort aptamers. It has been demonstrated that escort aptamers can be used to design efficient and highly specific systems for the delivery of therapeutic agents into cells, for the detection of cells of a definite type, for cell sorting, and for the selective blockage of surface proteins. Year after year, the number of studies devoted to the selection and application of escort aptamers against various cell targets (from bacteria to stem cells) steadily increases. It should be noted that the selection of aptamers against living cells has remained a more laborious and delicate process than the selection of aptamers against individual compounds. However, the increasing interest in this field and the advances in the selection methods are reasons to be hopeful that a greater variety of escort aptamers and aptamer-based therapeutic and diagnostic agents will appear in the near future. 

## Abbreviations

dsDNA: double-stranded DNA
ssDNA: single-stranded DNA
siRNA: small interfering RNA
PSMA: prostate-specific membrane antigen
IC_50_: therapeutical concentration required to suppress the growth of 50% cells
*K*_d_: apparent constant of dissociation of the aptamer–target complex
LNA: locked nucleic acids
SELEX: Systematic Evolution of Ligands by EXponential Enrichment

## References

[1] Ellington A.D., Szostak J.W. (1990). Nature..

[2] Tuerk C., Gold L. (1990). Science..

[3] Robertson D.L., Joyce G.F. (1990). Nature..

[4] Mayer G. (2009). Angew. Chem. Int. Ed..

[5] Stoltenburg R., Reinemann C., Strehlitz B. (2007). Biomol. Eng..

[6] Shamah S.M., Healy J.M., Cload S.T. (2008). Acc. Chem. Res..

[7] Zhou J., Rossi J.J. (2011). Oligonucleotides..

[8] Syed M.A., Pervaiz S. (2010). Oligonucleotides..

[9] Chapman J.A., Beckey C. (2006). Ann. Pharmacother..

[10] Ng E.W., Shima D.T., Calias P., Cunningham E.T., Guyer D.R., Adamis A.P. (2006). Nat. Rev. Drug. Discov..

[11] Hicke B.J., Stephens A.W. (2000). J. Clin. Invest..

[12] Breaker R.R. (1997). Curr. Opin. Chem. Biol..

[13] Fitzwater T., Polisky B. (1996). Meth. Enzymol..

[14] Sousa R. (2000). Meth. Enzymol..

[15] Chelliserrykattil J., Ellington A.D. (2004). Nat. Biotechnol..

[16] Burmeister P.E., Lewis S.D., Silva R.F., Preiss J.R., Horwitz L.R., Pendergrast P.S., McCauley T.G., Kurz J.C., Epstein D.M., Wilson C. (2005). Chem. Biol..

[17] Schmidt K.S., Borkowski S., Kurreck J., Stephens A.W., Bald R., Hecht M., Friebe M., Dinkelborg L., Erdmann V.A. (2004). Nucl. Acids Res..

[18] Shangguan D., Tang Z., Mallikaratchy P., Xiao Z., Tan W. (2007). ChemBiochem..

[19] Hicke B.J., Marion C., Chang Y.F., Gould T., Lynott C.K., Parma D., Schmidt P.G., Warren S. (2001). J. Biol. Chem..

[20] Morris K.N., Jensen K.B., Julin C.M., Weil M., Gold L. (1998). Proc. Natl. Acad. Sci. USA..

[21] Lupold S.E., Hicke B.J., Lin Y., Coffey D.S. (2002). Cancer Res..

[22] Ferreira C., Papamichael K., Guilbault G., Schwarzacher T., Gariepy J., Missailidis S. (2008). Anal. Bioanal. Chem..

[23] Ferreira C.S., Matthews C.S., Missailidis S. (2006). Tumour Biol..

[24] Ferreira C.S., Cheung M.C., Missailidis S., Bisland S., Gariepy J. (2009). Nucl. Acids Res..

[25] Daniels D.A., Chen H., Hicke B.J., Swiderek K.M., Gold L. (2003). Proc. Natl. Acad. Sci. USA..

[26] Shangguan D., Li Y., Tang Z., Cao Z.C., Chen H.W., Mallikaratchy P., Sefah K., Yang C.J., Tan W. (2006). Proc. Natl. Acad. Sci. USA..

[27] Shangguan D., Cao Z.C., Li Y., Tan W. (2007). Clin. Chem..

[28] Shangguan D., Cao Z., Meng L., Mallikaratchy P., Sefah K., Wang H., Li Y., Tan W. (2008). J. Proteome Res..

[29] Xiao Z., Shangguan D., Cao Z., Fang X., Tan W. (2008). Chemistry..

[30] Li N., Ebright J.N., Stovall G.M., Chen X., Nguyen H.H., Singh A., Syrett A., Ellington A.D. (2009). J. Proteome Res..

[31] Tang Z., Shangguan D., Wang K., Shi H., Sefah K., Mallikаratchy P., Chen H.W., Li Y., Tan W. (2007). Anal. Chem..

[32] Mallikaratchy P., Tang Z., Kwame S., Meng L., Shangguan D., Tan W. (2007). Mol. Cell. Proteom..

[33] Mallikaratchy P.R., Ruggiero A., Gardner J.R., Kuryavyi V., Maguire W.F., Heaney M.L., McDevitt M.R., Patel D.J., Scheinberg D.A. (2011). Nucl. Acids Res..

[34] Sefah K., Tang Z.W., Shangguan D.H., Chen H., Lopez-Colon D., Li Y., Parekh P., Martin J., Meng L., Phillips J.A. (2009). Leukemia..

[35] Chen H.W., Medley C.D., Sefah K., Shangguan D., Tang Z., Meng L., Smith J.E., Tan W. (2008). ChemMedChem..

[36] Shangguan D., Meng L., Cao Z.C., Xiao Z., Fang X., Li Y., Cardona D., Witek R.P., Liu C., Tan W. (2008). Anal. Chem..

[37] Blank M., Weinschenk T., Priemer M., Schluesener H. (2001). J. Biol. Chem..

[38] Zueva E., Rubio L.I., Ducongé F., Tavitian B. (2011). Int. J. Cancer..

[39] Mi J., Liu Y., Rabbani Z.N., Yang Z., Urban J.H., Sullenger B.A., Clary B.M. (2010). Nat. Chem. Biol..

[40] Kraus E., James W., Barclay A.N. (1998). J. Immunol..

[41] Davis K.A., Lin Y., Abrams B., Jayasena S.D. (1998). Nucl. Acids Res..

[42] Santuli-Marotto S., Nair S.K., Rusconi C., Sullenger B., Gilboa E. (2003). Cancer Res..

[43] Chen C.-H.B., Dellamaggiore K.R., Ouellette C.P., Sedano C.D., Lizadjohry M., Chernis G.A., Gonzales M., Baltasar F.E., Fan A.L., Myerowitz R. (2008). Proc. Natl. Acad. Sci. USA..

[44] Ohuchi S.P., Ohtsu T., Nakamura Y. (2006). Biochimie..

[45] Cerchia L., Duconge F., Pestourie C., Boulay J., Aissouni Y., Gombert K., Tavitian B., de Franciscis V., Libri D. (2005). PLoS Biol..

[46] Pestourie C., Cerchia L., Gombert K., Aissouni Y., Boulay J., De Franciscis V., Libri D., Tavitian B., Duconge F. (2006). Oligonucleotides..

[47] Wang C., Zhang M., Yang G., Zhang D., Ding H., Wang H., Fan M., Shen B., Shao N. (2003). J. Biotechnol..

[48] Guo K., Wendel H.P., Scheideler L., Ziemer G., Scheule A.M. (2005). J. Cell Mol. Med..

[49] Guo K.T., Schafer R., Paul A., Gerber A., Ziemer G., Wendel H.P. (2006). Stem Cells..

[50] Hoffmann J., Paul A., Harwardt M., Groll J., Reeswinkel T., Klee D., Moeller M., Fischer H., Walker T., Greiner T. (2008). J. Biomed. Mater. Res. A..

[51] Berezovski M.V., Lechmann M., Musheev M.U., Mak T.W., Krylov S.N. (2008). J. Am. Chem. Soc..

[52] Homann M., Göringer H.U. (1999). Nucl. Acids Res..

[53] Homann M., Göringer H.U. (2001). Bioorg. Med. Chem..

[54] Göringer H.U., Homann M., Zacharias M., Adler A. (2006). Handb. Exp. Pharmacol..

[55] Homann M., Lorger M., Engstler M., Zacharias M., Göringer H. (2008). Comb. Chem. High Throughput Screen..

[56] Göringer H.U., Homann M., Lorger M. (2003). Int. J. Parasitol..

[57] Lorger M., Engstler M., Homann M., Göringer H.U. (2003). Eukaryot. Cell..

[58] Ulrich H., Magdesian M.H., Alves M.J., Colli W. (2002). J. Biol. Chem..

[59] Chen F., Zhou J., Luo F., Mohammed A.B., Zhang X.L. (2007). Biochem. Biophys. Res. Commun..

[60] Torres-Chavolla E., Alocilja E.C. (2009). Biosens. Bioelectron..

[61] Ikanovic M., Rudzinski W., Bruno J., Allman A., Carrillo M., Dwarakanath S., Bhahdigadi S., Rao P., Kiel J., Andrews C. (2007). J. Fluoresc..

[62] Joshi R., Janagama H., Dwivedi H.P., Senthil Kumar T.M.A., Jaykus L.-A., Schefers J., Sreevatsan S. (2009). Mol. Cell. Probes..

[63] Pan Q., Zhang X.-L., Wu H.-Y., He P.-W., Wang F., Zhang M.-S., Hu J.-M., Xia B., Wu J. (2005). Antimicrob. Agents Chemother..

[64] Cao X., Li S., Chen L., Ding H., Xu H., Huang Y., Li J., Liu N., Cao W., Zhu Y. (2009). Nucl. Acids Res..

[65] Hamula C.L.A., Zhang H., Guan L.L., Li X.-F., Le X.C. (2008). Anal. Chem..

[66] Bruno J., Carrillo M., Phillips T., Andrews C. (2010). J. Fluoresc..

[67] So H.-M., Park D.-W., Jeon E.-K., Kim Y.-H., Kim B.S., Lee C.-K., Choi S.Y., Kim S.C., Chang H., Lee J.-O. (2008). Small..

[68] Dwivedi H., Smiley R., Jaykus L.-A. (2010). Appl. Microbiol. Biotechnol..

[69] Barfod A., Persson T., Lindh J. (2009). Parasitol. Res..

[70] Chen F., Hu Y., Li D., Chen H., Zhang X.-L. (2009). PLoS One..

[71] Chu T.C., Marks J.W., Lavery L.A., Faulkner S., Rosenblum M.G., Ellington A.D., Levy M. (2006). Cancer Res..

[72] Bagalkot V., Farokhzad O.C., Langer R., Jon S. (2006). Angew. Chem. Int. Ed. Engl..

[73] Huang Y.F., Shangguan D., Liu H., Phillips J.A., Zhang X., Chen Y., Tan W. (2009). ChemBiochem..

[74] Chu T.C., Twu K.Y., Ellington A.D., Levy M. (2006). Nucl. Acids Res..

[75] McNamara J.O., Andrechek E.R., Wang Y., Viles K.D., Rempel R.E., Gilboa E., Sullenger B.A., Giangrande P.H. (2006). Nat. Biotechnol..

[76] Dassie J.P., Liu X.-Y., Thomas G.S., Whitaker R.M., Thiel K.W., Stockdale K.R., Meyerholz D.K., McCaffrey A.P., McNamara J.O., Giangrande P.H. (2009). Nat. Biotechnol..

[77] Wullner U., Neef I., Eller A., Kleines M., Tur M.K., Barth S. (2008). Curr. Cancer Drug Targets..

[78] Khaled A., Guo S., Li F., Guo P. (2005). Nano Lett..

[79] Zhou J., Swiderski P., Li H., Zhang J., Neff C.P., Akkina R., Rossi J.J. (2009). Nucl. Acids Res..

[80] Huang Y.F., Chang H.T., Tan W. (2008). Anal. Chem..

[81] Huang Y.F., Sefah K., Bamrungsap S., Chang H.T., Tan W. (2008). Langmuir..

[82] Farokhzad O.C., Jon S., Khademhosseini A., Tran T.N., Lavan D.A., Langer R. (2004). Cancer Res..

[83] Farokhzad O.C., Cheng J., Teply B.A., Sherifi I., Jon S., Kantoff P.W., Richie J.P., Langer R. (2006). Proc. Natl. Acad. Sci. USA..

[84] Cheng J., Teply B., Sherifi I., Sung J., Luther G., Gu F.X., Levy-Nissenbaum E., Radovic-Moreno A.F., Langer R., Farokhzad O.C. (2007). Biomaterials..

[85] Farokhzad O.C., Khademhosseini A., Jon S., Hermmann A., Cheng J., Chin C., Kiselyuk A., Teply B., Eng G., Langer R. (2005). Anal. Chem..

[86] Wu Y., Sefah K., Liu H., Wang R., Tan W. (2010). Proc. Natl. Acad. Sci. USA..

[87] Zhou J., Soontornworajit B., Martin J., Sullenger B.A., Gilboa E., Wang Y. (2009). Macromol. Biosci..

[88] Perkins A.C., Missailidis S. (2007). Q. J. Nucl. Med. Mol. Imaging..

[89] Hicke B.J., Stephens A.W., Gould T., Chang Y.-F., Lynott C.K., Heil J., Borkowski S., Hilger C.-S., Cook G., Warren S. (2006). J. Nucl. Med..

[90] Chu T.C., Shieh F., Lavery L.A., Levy M., Richards-Kortum R., Korgel B.A., Ellington A.D. (2006). Biosens. Bioelectron..

[91] Min K., Song K.-M., Cho M., Chun Y.-S., Shim Y.-B., Ku J.K., Ban C. (2010). Chem. Commun..

[92] Javier D.J., Nitin N., Levy M., Ellington A., Richards-Kortum R. (2008). Bioconjugate Chem..

[93] Chen Y., Munteanu A.C., Huang Y.-F., Phillips J., Zhu Z., Mavros M., Tan W. (2009). Chem. Eur. J..

[94] Terazono H., Anzai Y., Soloviev M., Yasuda K. (2010). J. Nanobiotechnol..

[95] Liu G., Mao X., Phillips J.A., Xu H., Tan W., Zeng L. (2009). Anal. Chem..

[96] Pai S.S., Ellington A.D. (2009). Meth. Mol. Biol..

[97] Zelada-Guillén G.A., Jordi R., Düzgün A., Rius F.X. (2009). Angew. Chem. Int. Ed..

[98] Lee H.-J., Kim B.C., Kim K.-W., Kim Y.K., Kim J., Oh M.-K. (2009). Biosens. Bioelectron..

